# The complete chloroplast genome of *Dryopteris crassirhizoma* Nakai

**DOI:** 10.1080/23802359.2021.1933634

**Published:** 2021-05-27

**Authors:** Qi Wang, Wenting Xu, Lirong Zhou, Biwei Mai, Naiyun Zhu, Xiaoli Zhao, Zhixian Lei

**Affiliations:** a Department of Critical Medicine, Hainan Maternal and Children’s Medical Center, Haikou, P.R. China; b Department of Chinese medicine, Hainan Maternal and Children’s Medical Center, Haikou, P.R. China

**Keywords:** *Dryopteris crassirhizoma*, chloroplast genome, phylogenetic analysis, Dryopteridaceae

## Abstract

*Dryopteris crassirhizoma* Nakai is a fern plant with important evolutionary and medicinal values. Herein, we assembled the complete chloroplast genome of *D. crassirhizoma* by next-generation sequencing technology. The complete chloroplast genome of *D. crassirhizoma* was 153,355 bp in length, and the GC content was 42.86%; the genome consisted of a pair of inverted repeats (IRs, 23,470 bp), a small single copy region (SSC, 21,570 bp) and a large single copy region (LSC, 84,854 bp). The genome contained 111 genes, namely, 73 protein-coding genes, 34 tRNA genes and four rRNA genes. The phylogenetic analysis suggested that both *D. crassirhizoma* and *D. decipiens* from Dryopteridaceae were most closely related to *Lepisorus clathratus* from Polypodiaceae.

*Dryopteris crassirhizoma* Nakai is a fern plant classified into the Dryopteridaceae family (Sessa et al. 2012). The dry rhizomes and petiole residues of this plant is a traditional Chinese herbal medicine used for the treatment of diseases such as fever, cancer, ancylostomiasis and other parasitosis. In particular, this herb was used to cure severe acute respiratory syndrome (SARS) and bird flu (Zhao et al. 2007; Wang et al. 2017). Previous studies have demonstrated that special active ingredients from its dried rhizome show potential pharmacological activities (Wang et al. 2017; Chen et al. 2020), but limited genomic and genetic resources have impeded the precise identification of *D. crassirhizoma* until now. Herein, we generated the whole chloroplast genome of *D. crassirhizoma*, which will provide useful informative data for further research on molecular identification of *D. crassirhizoma* and evolution of fern plants.

DNA was isolated *via* the modified CTAB method from the fresh leaves of an individual *D. crassirhizoma* plant in the greenhouse at Chengxi District (110°19.245′E, 19°59.757′N), Haikou, China (Ding et al. 2020), and a specimen was deposited at the herbarium of Hainan Maternal and Children's Medical Center (http://www.hnwcmc.com/, Qi Wang, wqi1220@163.com) under voucher number 11_1. The DNA library was constructed with the NEBNext Ultra^TM^ DNA library Prep Kit (New England Biolabs, Ipswich, MA) and then sequenced on the Illumina NovaSeq platform (Illumina, CA, USA). The raw data were filtered using SOAPnuke v1.3.0 with default settings (Chen et al. 2018), and the paired-end reads of the cleaned data were assembled into circular contigs using SPAdes v3.13.0 with the parameter -k 127 (Bankevich et al. 2012). The final draft cp genome was corrected using GapCloser v 1.12 (Xu et al. 2020). The annotation was performed using PGA (Qu et al. 2019) and then submitted to GenBank (accession no. MW557379).

This complete chloroplast genome of *D. crassirhizoma* was 153,355 bp in length and consisted of a small single copy region (SSC) of 21,570 bp, a large single copy (LSC) region of 84,854 bp and a pair of inverted repeat (IR) regions of 23,470 bp. This cp genome showed an overall GC content of 42.86%, whereas the corresponding GC contents in the SSC, LSC and IR regions were 41.83%, 40.25% and 45.37%, respectively. Genome annotation indicated the presence of 111 full-length genes, including 73 protein-coding genes with an average length of 597.08 bp, 34 transfer RNA genes with an average length of 75.31 bp and four ribosomal RNA genes with an average length of 1123.5 bp. Seven genes (*atpF*, *matK*, *ndhA*, *petA*, *rpoC1*, *rps12*, *rps16*) contained a single intron, and two genes (*clpP*, *ycf3*) contained two introns, whereas eight genes (*rps12*, *rrn5*, *rrn4*, *rrn23*, *rrn16*, *trnA-UGC*, *trnG-UCC*, *trnL-UAA*) had two copies in this cp genome.

Phylogenetic analysis was performed with the neighbor-joining (NJ) method in MEGA X based on 14 complete cp genomes of ferns, including *D. crassirhizoma* (Kumar et al. 2018). The results revealed that *D. crassirhizoma* was classified into the genus of *Dryopeteris* from Dryopteridaceae with high bootstrap support values, indicating that the genus of *Dryopteris* has a closer evolutionary relationship with the genus of *Lepisorus* than with the genera of *Athyrium*, *Diplazium*, *Rhachidosorus* and *Cystopteris* (Figure 1). This finding is consistent with a previous study of phylogenetic trees in Dryopteridaceae and Polypodiaceae (Wang et al. 2019).

**Figure 1.**
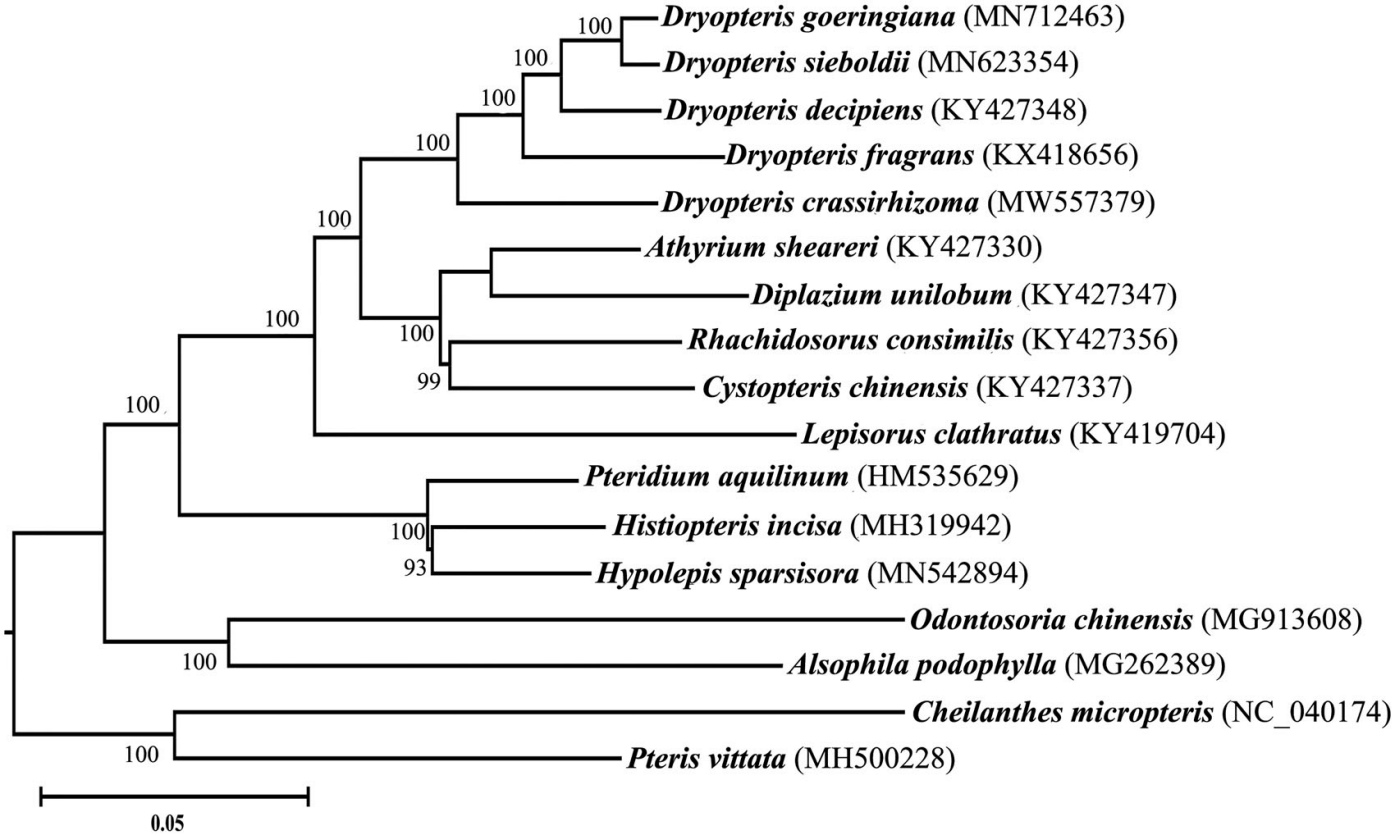
NJ phylogenetic tree based on 17 species chloroplast genomes was constructed using MEGA X.

## Data Availability

The data that support the findings of this study are openly available in the US National Center for Biotechnology Information (NCBI database) at https://www.ncbi.nlm.nih.gov/, reference number: MW557379. The associated BioProject, BioSample and SRA numbers are PRJNA692559, SAMN17348916, SRR13447701 and SSR13447702.
